# Open hemorrhoidectomy under local anesthesia versus saddle block in western Uganda: a study protocol for a prospective equivalence randomized, double-blind controlled trial

**DOI:** 10.1186/s13063-022-06636-8

**Published:** 2022-08-13

**Authors:** Franck Katembo Sikakulya, Robinson Ssebuufu, Xaviour Francis Okedi, Moris Baluku, Herman Lule, Patrick Kyamanywa

**Affiliations:** 1 Faculty of Clinical Medicine and Dentistry, Department of Surgery, Kampala International University Western Campus, Ishaka-Bushenyi, Uganda; 2 Faculty of Medicine, Université Catholique du Graben, Butembo, Democratic Republic of the Congo; 3 Uganda Medical and Dental Practitioners Council, Kampala, Uganda; 4 Department of Anesthesia and critical care, Kabale University, Kabale, Uganda; 5 Department of Surgery, Kiryandongo Hospital, Kiryandongo, Uganda; 6 Uganda Martyrs University, Nkozi, Uganda

**Keywords:** Third- or 4th-degree hemorrhoids, Local anesthesia, Saddle block, Open hemorrhoidectomy, Uganda

## Abstract

**Background:**

While open hemorrhoidectomy under local anesthesia has been shown to be more cost-effective with shorter operation times and lower complication rates, local anesthesia is still not considered as a first-line technique in low-income countries like Uganda. The objective of this trial is to compare open hemorrhoidectomy using local anesthesia versus saddle block among patients with primary uncomplicated 3rd- or 4th-degree hemorrhoids in western Uganda.

**Methods:**

The protocol for a prospective equivalence randomized, double-blind controlled trial was conducted among patients with primary uncomplicated 3rd- or 4th-degree hemorrhoids. Recruitment was started in December 2021 and is expected to end in May 2022. Consenting participants who require open hemorrhoidectomy indicated at Kampala International Teaching Hospital, Uganda, will be randomized into two groups of 29 patients per arm.

**Discussion:**

The primary outcome of this study is to compare the occurrences of postoperative pain following open hemorrhoidectomy using the visual analog scale in an interval of 2, 4, and 6 h and 7 days postoperatively. Furthermore, the mean operative time from the induction of anesthesia to the end of the surgical procedure as well as the cost-effectiveness of the 2 techniques will be assessed in both groups. Open hemorrhoidectomy under local anesthesia has the potential to offer benefits to patients but most importantly expediting return to baseline and functional status, shorter hospital stay by meeting the faster discharge criteria, and reduction in costs associated with reduced length of stay and complications.

**Trial registration:**

Pan African Clinical Trials Registry PACTR202110667430356. Registered on 8 October 2021

## Introduction

Hemorrhoids, the most prevalent anal disease, are defined as collections of submucosal, fibrovascular, arterio-venous sinusoids that are part of the anorectum [1]. Clinically, hemorrhoids are expressed by bright red bleeding from the rectum with mucous discharge, perianal irritation and pain, prolapse of the hemorrhoidal cushions, bulging masses, soiling, and non-hygiene [1]. The paradigm about the treatment of hemorrhoids has changed over the years, and many options have been reported about hemorrhoid surgery [2]. These options range from conservative treatment (dietary and sclerotherapy) to surgical methods like band ligation and excision according to the grade of hemorrhoids [2, 3]. The classification by Banov L et al., grade III and IV hemorrhoids are amenable to surgical treatment, and open hemorrhoidectomy is effective and seems to be the most common technique for grade III and IV hemorrhoids [4, 5]. Surgical treatment is the only truly curative method of hemorrhoidal disease. This is indicated in patients to whom conservative measures have failed and for those who have developed complications. Of the several surgical techniques, the Milligan-Morgan hemorrhoidectomy is still considered the treatment of choice, since it is the most radical one and it has the best results [6]. One of the major problems associated with the technique remains postoperative pain. Studies reported severe pain occurred in 20–40% of patients [6, 7].

Local anesthesia (LA) is adequate for the majority of anal surgical procedures [8]. LA produces sensory and motor blockade in the peri-anal region with an effect on peripheral nerve endings and therefore produces varying and unpredictable degrees of anal canal relaxation [8]. Operative time has been shown to be different according to the anesthetic technique used in terms of time saved during operation according to studies done by Kushwaha and Baghel with their colleagues comparing the use of LA versus spine and GA for open hemorrhoidectomy [2, 9]. Despite producing interesting results presented in the literature, LA has been increasingly questioned, as to whether it brings more benefits to selected patients [10]. It was found that open hemorrhoidectomy (OH) under general anesthesia (GA) or spine anesthesia (SA) showed a high-cost implication as compared to OH done under local anesthesia in different studies published [2, 10, 11].

Open hemorrhoidectomy in most low- and middle-income countries (LMICs) is performed under saddle block which requires a trained anesthetic provider and is associated with delays in the initiation of surgery, postoperative urinary retention, neural injury, direct nerve and spinal cord injury, cauda equina syndrome, epidural hematoma post-dural puncture headache, failed block, epidural abscess, and hematoma [2, 12, 13]. These complications increase the duration of hospital stay and morbidity [13]. The few numbers of anesthetic providers in developing countries like Uganda (0.05 per 100,000 population compared to 17.85 per 100,000 in the UK [14]) have been seen as a hindrance to access and performance of open hemorrhoidectomy especially at low-level facilities despite open hemorrhoidectomy being a very common surgical procedure [8]. The costs of anesthetic procedures have become an important factor in the selection of the best technique for benign anal surgeries [10]. Saddle block needs a trained anesthetist to administer the anesthesia in order to perform open hemorrhoidectomy and has been associated with a long hospital stay [2, 13] which increases the cost related to OH compared to OH done under local anesthesia [2, 3, 9, 10].

While open hemorrhoidectomy under local anesthesia has been shown to have lower complication rates and more cost-effective by saving anesthetics for other surgeries, it has been also found to increase patient turnover because of the shorter operative time [2, 7]. In spite of these benefits, LA is still not considered as a first-line technique in low-income countries like Uganda. To explore the applicability of LA for OH in a limited setting, a well-designed randomized controlled trial is needed.

## Study rationale

Sound knowledge of safe and cheaper surgical options is mandatory to inform policy decisions in a low-income country like Uganda where the health care system is already constrained by other surgical and obstetric emergencies. This study will not only be a benchmark for future research but will also influence future trends in managing uncomplicated 3rd- and 4th-degree hemorrhoids in low-income countries.

## Main objective

The main objective is to compare open hemorrhoidectomy using local anesthesia versus saddle block among patients with primary uncomplicated 3rd- or 4th-degree hemorrhoids in western Uganda.

## Specific objectives

The following are the specific objectives:

i. To compare the occurrence of postoperative pain following open hemorrhoidectomy under local anesthesia versus open hemorrhoidectomy under saddle block
ii. To compare the mean operative time for open hemorrhoidectomy under local anesthesia versus open hemorrhoidectomy under saddle block
iii. To compare the cost-effectiveness between open hemorrhoidectomy under local anesthesia and saddle block

## Hypothesis

There is no significant difference in the surgical outcome between the use of local anesthesia versus saddle block for open hemorrhoidectomy of uncomplicated 3rd- or 4th-degree hemorrhoids.

## Methodology

### Study design

This study will be a prospective equivalence randomized, double-blind controlled trial conducted in the department of surgery at Kampala International University Teaching Hospital (KIU-TH), western Uganda.

### Study area

The study will be conducted in KIU-TH in the operating theater and general surgery ward.

KIU-TH is a private nonprofit teaching hospital of Kampala International University located in Bushenyi district, south-western Uganda about 370 km from Kampala, the capital city of Uganda. The hospital has a bed capacity of 700. The hospital consists of outpatient departments (general and special), inpatient departments for surgery (general surgery and orthopedic), gynecology and obstetrics, internal medicine, and pediatrics. The department has five theater rooms with an average of 800 elective surgeries per year. The Department of General Surgery has 13 surgeons and 42 residents. There are 3 days per week for elective surgery. There are three anesthesiologists and six anesthetists. KIU-TH has a 4-bed intensive care unit (ICU) being managed by anesthesiologists.

### Study population

All patients were admitted to the surgical ward for elective hemorrhoidectomy during the study period.

#### Inclusion criteria

All patients:

- Aged between 18 and 65 years
- Uncomplicated 3rd- and 4th-degree hemorrhoids
- Classified by the American Society of Anesthesiologists (ASA) as I and II
- Mixed hemorrhoids with a bulging external component

#### Exclusion criteria

All patients with:

- Contraindication of spinal anesthesia (chronic backache and spinal deformities)
- Previous perianal surgery
- A known history of allergy to local anesthetics
- Bleeding disorders
- Third- and 4th-degree thrombosed hemorrhoids
- Active inflammatory bowel disease
- Pregnant women
- Documented neoplasm situated distal aspect of the large bowel or rectum
- Liver diseases with portal hypertension
- Failed anesthetic technique

### Sample size determination

The randomized control trial assumes a null hypothesis that the mean postoperative pain scores after hemorrhoidectomy using local anesthesia are not different from that done under saddle block by a clinically relevant amount. Since the primary outcome measure was a continuous variable (i.e., pain level measured using a visual analog scale), the formula for an equivalence design in randomized control trials by Zhong et al. [15] was used.

$$\boldsymbol{N}=\mathbf{2}\times {\left\{\frac{{\boldsymbol{Z}}_{\mathbf{1}-\propto}+{\boldsymbol{Z}}_{\mathbf{1}-\boldsymbol{\beta}}}{\boldsymbol{\delta}}\right\}}^{\mathbf{2}}\times {\boldsymbol{S}}^{\mathbf{2}}$$

where

***N***sample size per group

***Z***_***1−α***_standard normal deviate for a two-sided test used in equivalence trials (1.96 for 95% confidence interval)

***δ***clinically admissible margin of equivalence design as a difference

***α***type I error associated with rejecting the null hypothesis when it is true; (0.05) for 95% confidence interval

***β***type II error associated with the alternative hypothesis, assumed to be 0.20 for a statistical power of 80%

**(1−*β*)**the probability of rejecting the null hypothesis when it is false, i.e., *Z*_1−*β*_ = 0.845 for statistical power of 80%

***S***^**2**^pooled standard deviation of both comparison groups

According to a similar randomized control trial that compared open hemorrhoidectomy under local anesthesia versus supine anesthesia at the Korle Bu Teaching Hospital in Ghana [16], the difference in the mean pain scores between the 2 groups was *δ* = 0.73; pooled standard deviation in the mean pain scores *S* = 1.9 (average of 2.356 for local anesthesia and 1.479 for supine anesthesia), an estimated sample size of 104 participants per group, was obtained. Since at the time of the proposed study, nothing was known about the mean pain score following OH under local anesthesia and the real difference in clinical outcome between the two techniques in Uganda, the sample size was adjusted using Slovin’s formula as detailed by Ellen [17] based on the hospital records, Kampala International University Teaching Hospital which registered an average of 33 cases of grade 3 or 4 hemorrhoids in a period of 6 months that corresponded to the intended data collection period; thus, a sample size of 26 participants per group was obtained which was increased to 29 participants in each arm, based on a rate of 10% in each arm to compensate for the loss to follow-up and non-responsiveness found.

### Study variables

#### Primary outcome variable

The occurrence of pain following open hemorrhoidectomy will be determined in both groups using the area under the curve (AUC) for visual analog scale (VAS). The VAS will be used in an interval of 2, 4, and 6 h and 7 days postoperatively (the lower end of the scale labeled “0” means no pain while the upper end of the scale “10” signifies the worst imaginable pain).

#### Secondary outcome variable

The mean operative time from anesthesia induction to the end of open hemorrhoidectomy will be recorded in both groups.

#### Tertiary outcome variable

The tertiary outcome is the cost-effectiveness of the 2 surgical approaches in relation to the amount of materials used in the 2 arms.

#### Independent variables

Data will be captured on preoperative independent variables such as gender, age, ASA level, and degrees of the hemorrhoids.

### Consent

Any patient with third- and/or fourth-degree hemorrhoids aged above 18 years is eligible for this study and will be given an equal chance to participate and will be consented to by the principal investigator at the time of screening for eligibility.

### Randomization and blinding

After fulfilling the enrollment criteria and obtaining the informed consent, the participants will be allocated randomly by assistant researchers well trained to either the local anesthesia group (group A) or the saddle block group (group B) by using envelope allocation concealment whereby half of the envelopes will contain a chit with letter A while the other half will have a chit with letter B signifying local anesthesia and saddle block, respectively. The content of the envelope will be opened once the patient is in the theater, and the patients and outcome assessors will be blinded about the anesthesia.

#### Participant recruitment and study procedure

Participants will be recruited from surgical outpatient by trained research assistants. Patients with uncomplicated 3rd- or 4th-degree hemorrhoids will be assessed for study eligibility using a screening log. Written informed consent will be obtained from the eligible participants.

The patients admitted for elective hemorrhoidectomy will undergo pre-anesthetic evaluation the day before surgery and will be given fasting guidelines including 6 h for solid foods and 2 h to anesthesia for clear fluids like water. All patients will receive prophylactic antibiotics (intravenous 500 mg of metronidazole) within 60 min prior to incision [18]. Two hours before surgery, the patient will be receiving an enema intrarectal. All patients scheduled for surgery will be informed and admitted a day prior to surgery, prepared, and included in the theater program in order to maintain continuity of care.

Once in the operating room, vital signs will be taken (blood pressure, pulse rate, oxygen saturation, and monitoring of the ECG). Co-loading of intravenous crystalloids, namely normal saline and or ringers’ lactate, will be done using a restrictive approach of fluid administration and undergo open hemorrhoidectomy under saddle block [19].

**Fig. 1:**
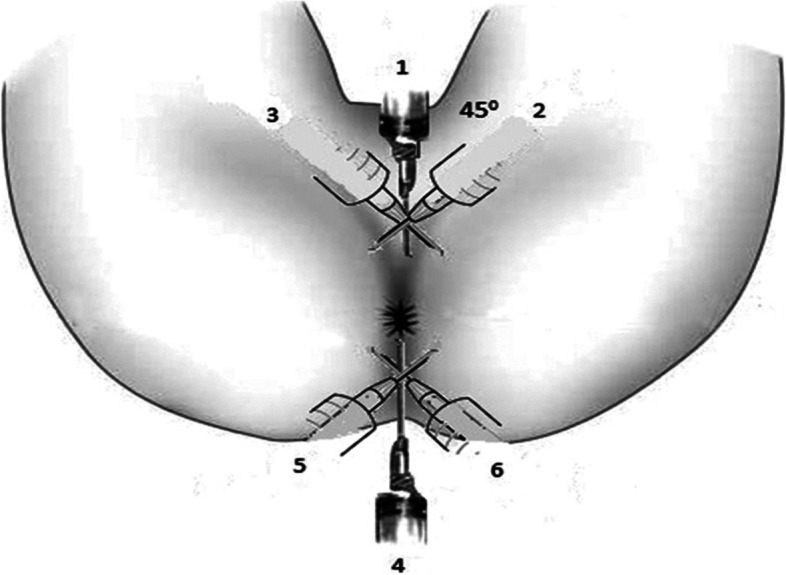
Local anesthesia infiltration [20]

Aseptic protocols will be observed for all groups. Group A patients, in lithotomy or prone jack-knife positions, will be infiltrated with bupivacaine 0.5% at a maximum safe dose of 2 mg/kg with adrenaline (1:200,000) following the technique of Jinjil et al. [20]. The local anesthetic mixture will be administered by the principal investigator as follows: The first two injections will be applied bilaterally (1 and 4), 5 mm from the border of the perianal skin, and the mixture will be injected at 12 and 6 o’clock (3 ml each side). Thereafter, further 4 injections will be administered into 4 quadrants performing a diamond shape (2, 3, 5, and 6), 5 mm from the anal opening; every 1 ml will be injected at a 4-cm, 3-cm, and 2-cm depth from the skin, respectively, as shown in Fig. 1.

With this technique, the mixture will fill the inter-sphincteric space and block the inferior rectal branch of the pudendal nerve (S2, S3) and the perineal branch of the 4th sacral nerve, causing paralysis of the external sphincter.

For group B patients, the back will be cleaned with 70% alcohol and draped while in a sitting position. The anesthetist or the anesthesiologist will identify the L4/L5 interspace by anatomical landmarks. Lidocaine will be infiltrated in the skin and subcutaneous tissue to form a wheel in the skin. A 25-G quincke spinal needle will be introduced into the sub-arachnoid space using either the midline or paramedian approach as deemed necessary by the anesthetist. Once the free flow of cerebrospinal fluid is observed in the hub of the needle, 1.5 ml of 0.5% bupivacaine will be administered. The patient will be kept in the sitting position for 10 min and thereafter placed in supine. Time 0 will be reordered once the cleaning of the back starts.

After the proof of the effect of anesthesia is administrated, participants in both groups will be put in lithotomy position, and the perianal region will be cleaned and draped before the beginning of open hemorrhoidectomy which will be done by the principal investigator. The technique for open hemorrhoidectomy will be as described by Milligan Morgan Clinic [21] after which dressing will be done, and this will be documented as a time finale.

Analgesia will be given according to the visual analog scale (VAS) once it is rated more than 4 by the patient at 2, 4, and 6 h for all patients irrespective of the type of anesthesia, and all participants will be discharged based on the post-anesthesia discharge scoring system (PADS) for determining home-readiness whereby the patient is judged fit for discharge when his score is ≥ 9 [22] and the 7th day assessment will be done after the patient is called back by the research assistants. Participants will be reassessed using the VAS on the 7th day postoperative. Diclofenac sodium 100 mg oral 8 hourly for 5 days will be considered as rescue analgesia postoperatively. All patients will be receiving a tablet of metronidazole 400 mg 8 hourly for the following five postoperative days.

The follow-up research assistants different from the recruiting team will collect the outcome data from patients at a stipulated time and enter it into a Microsoft Excel sheet up to the 7th day postoperative.

On the 7th day postoperative, the patients will be called back for review and follow-up assessment. Those who will not be able to come back to the hospital after discharge will be interviewed on a telephone call.

**Fig. 2:**
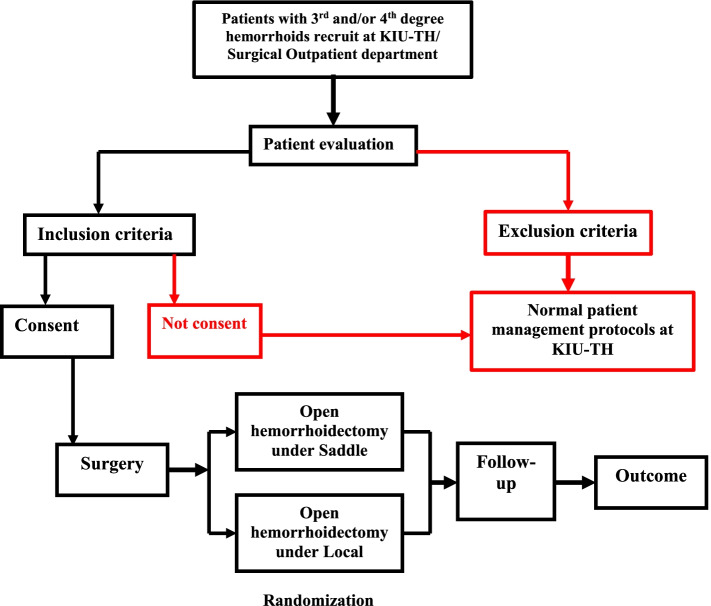
Flow chart of the trial process from allocation to follow-up at Kampala International University Teaching Hospital

**Fig. 3:**
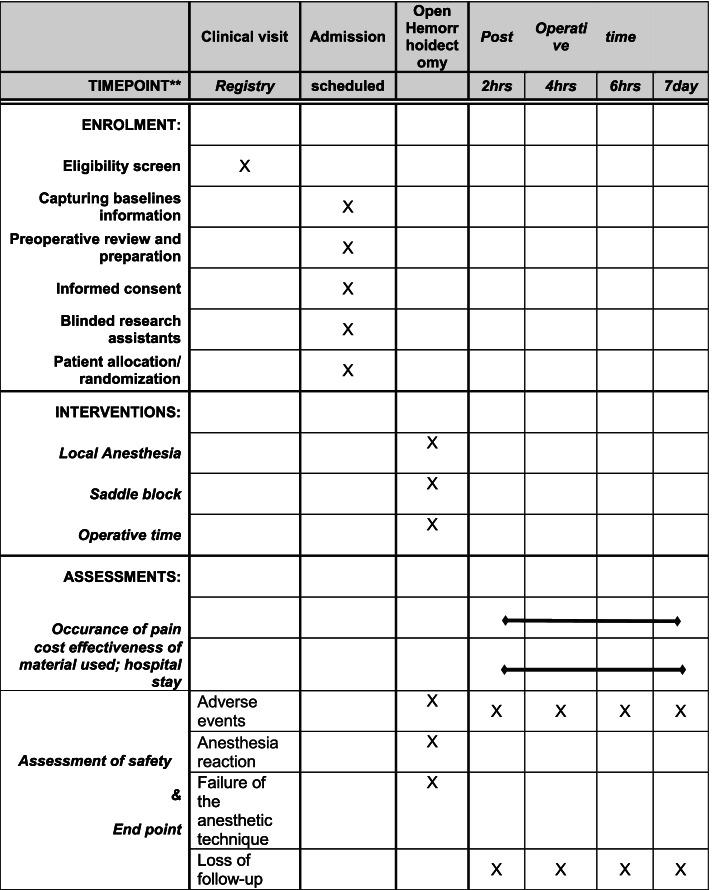
Standard Protocol Items: Recommendations for Interventional Trials (SPIRIT) figure of the trial schedule for enrollment, interventions, and follow-up/assessments at Kampala International Teaching Hospital

During both visits, detailed information will be obtained by the study doctor. The entire process from patient allocation to follow-up is displayed in Fig. 2 and well described in a Standard Protocol Items: Recommendations for Interventional Trials (SPIRIT) figure in Fig. 3.

### Interim analysis

Biostatisticians will perform an interim analysis once we register a total of 36 patients for open hemorrhoidectomy using the two different anesthetic techniques, which is 62.1% of the planned number of patients. The primary, secondary, and tertiary outcomes will be evaluated to compare open hemorrhoidectomy using local anesthesia versus saddle block among patients with primary uncomplicated 3rd- and 4th-degree hemorrhoids.

The Data Monitoring Committee would recommend continuing, discontinuing, or modifying the trial, if concerns regarding the effect and safety of the participants will arise.

The recommendation to withdraw a patient from the trial will be decided by the principal investigator in case of failure of the anesthetic technique and loss of follow-up.

A recommendation of trial termination would be considered by the principal investigator, clinical trial institution, and ethics committee if achieving the difference in treatment effects is unlikely or if intolerable adverse effects occurred. The detailed results remain confidential to the investigator. The interim analysis was done on 10 March 2022, and continued recruitment was recommended.

### Data processing and analysis plan

Data will be statistically analyzed using IBM Statistics SPSS for Windows 23.0. The primary outcome will analyze all randomized patients (on an intent-to-treat (ITT) basis) and per-protocol (PP) population. The mean pain scores at rest and their standard deviations will be computed and compared using one-way ANOVA tests. VAS will be stratified as ordinal (0 as no pain, 1–3 as mild, 4–6 as moderate, and 7–10 as severe); the significance of the difference in the mean scores between local and saddle groups will be determined by the Kruskall-Wallis (*H*) test at 95% confidence interval, regarding *p* < 0.05 as statistically significant; furthermore, the mean pain scores will be analyzed for significant differences in the area under the curve (AUC) for VAS (2-sample *t*-test). The mean operative time and standard deviation will be computed for each technique of open hemorrhoidectomy. Cross tabulation will be performed between the two open hemorrhoidectomy techniques for categorical cost-effective analysis. The difference in the means will be compared using the *t*-test and their corresponding two-tailed p-value, regarding *p* < 0.05 as statistically significant.

### Study duration

The study duration is a 6-month study period from 1 December 2021 to 31 May 2022. During this period, all participants will be followed up for a period of 7 days post-surgery. At the end of this trial, the result will be published in a peer-reviewed journal, and deidentified data set will be publically available to other researchers on a permanent web link that will be provided by a peer-reviewed journal.

### Trial status

The first case was recruited on 2 December 2021, and as of 7 April 2022, 45 patients were registered out of the required sample size of 58 for this protocol version 3 as of 21 May 2022.

#### Ethical considerations

This clinical trial has been approved by the Kampala International University Research Ethics Committee (KIU-REC) under the number KIU-REC-2021-24. The standard official consent form for the Kampala International University Research and ethical review committee will be adopted. Respective hospital surgical consent forms conforming to WHO consenting information standards will be used, and oral translation to the local language will be done by the researcher or his delegated assistant. Participant recruitment will be done non-discriminatively regardless of race, color, or tribe. All those who meet the selection criteria will have equal chances of participating. The inclusion and exclusion criteria will be followed strictly. The trial will be overseen by the Institutional Research Ethics Committee (IREC) of Kampala International University.

### Adverse events

There are minor discomforts anticipated in this study: pain associated with venipuncture during the process of blood collection for investigations which will be necessary as routine patient preparation for surgery. Patients are then expected to undergo pain and stress of hemorrhoids operation and bleeding after the operation. These discomforts are not imposed by the study but rather expected routine from patients who undergo such operations.

### Ancillary and post-trial care

The outcome of the study may indirectly help participants to obtain care during study time and will be recognized during publications. Diverse outcomes, e.g., too much pain, will result in converting from local to SA (crossover) and getting other analgesics other than those prescribed in the trial.

### Trial discontinuation

The trial will be discontinued for individual patients due to non-adherence, e.g., not completing the specified duration of follow-up, diverse outcomes, e.g., too much pain resulting in converting from local to GA (cross over) and getting other analgesics other than those prescribed in the trial.

### Unblinding

The recommendation to withdraw a patient from the trial will be decided by the principal investigator in case of failure of the anesthetic technique and loss of follow-up. In addition to that, outcome assessors will be blinded, and the criterion for unblinding is if they observe adverse effects related to intervention that needs to be disclosed to the attending clinician.

### Missing data

Missing data will be reported as missing during the analysis. Clinical characteristics of those lost to follow-up and crossovers will be compared to those retained in both groups.

## Discussion

### Study strengths

Open hemorrhoidectomy under local anesthesia has the potential to offer benefits to patients including expediting return to baseline and functional status, shorter hospital stay by meeting the discharge criteria faster, reduction in costs because of reduced length of stay, and reducing complications. The postoperative occurrence of pain, the main operative time, and cost-effectiveness will be assessed and reported for the first 7 days. This study is a randomized clinical trial that will provide level 1 evidence for evidence-based clinical practice about short-term surgical outcomes in open hemorrhoidectomy using an affordable alternative of low-cost technique in LMICs.

### Study limitations

Lack of cooperation or withdrawal of consent by some participants during the study will be managed by doing a comprehensive counseling of participants with regard to the participation. The formula produces a ballpark figure to work with and lacks statistical power of obtaining the error of tolerance according to Ryan (2013). No similarity in the price of materials used during the study will be settled by the use of prices from the Joint Medical Store of Uganda to standardize the prices.

The error of tolerance was obtained from a set error margin for a confidence interval of 95 using tables, to increase the power of precision in sample size determination occurrence. Targeted population computation was done from reliable sources of HMIS data based on the Ugandan Ministry of Health for the 3 months for KIU-TH to get the average population per month. The sample size has been adjusted to cater for loss to follow-up.

### COVID-19 standard operating procedures (SOPs)

To prevent the COVID-19 infection, we shall ensure compliance to regular hand washing or sanitizing, use of face masks, maintaining social distance, and disease screening at triage point to all patients and research team according to June 2020 Uganda Ministry of Health or as updated guidelines for COVID-19 management. We shall comply with the Uganda National Guidelines for Conduct of Research during the COVID-19 pandemic to ensure the safety of all research teams and participants in research activities.

## Data Availability

Not applicable.

## Abbreviations

ASA: American Society of Anesthesiologists
GA: General anesthesia
KIU-TH: Kampala International University Teaching Hospital
L.A: Local anesthesia
OH: Open hemorrhoidectomy
S.B: Saddle block
VAS: Visual analog scale

## References

[1] Jacobs D (2014). Hemorrhoids. N Engl J Med.

[2] Baghel PS, Joleya M, Suryavanshi S (2016). Comparison of open hemorrhoidectomy under local and spinal anesthesia and its practical feasibility at a tertiary care institute. IJSS J Surg.

[3] Burch J, Epstein D, Sari AB, Weatherly H, Jayne D, Fox D, Woolacott N, Morgan M (2009). Stapled haemorrhoidopexy for the treatment of haemorrhoids: a systematic review. Colorectal Dis.

[4] Younes HEA, Metwally YH, El-hussainy AF, Elsayed ME, Ahmad MS (2014). Local anesthesia versus spinal anesthesia for hemorrhoidectomy. AAMJ.

[5] Khubchandani IT, Bub DS. Traditional Hemorrhoidectomy: Techniques and Results. In: Ratto C, Parello A, Litta F (eds) Hemorrhoids. Coloproctology, vol 2. Cham: Springer; 2018. 10.1007/978-3-319-53357-5_19.

[6] Medina-Gallardo A, Curbelo-Peña Y, De Castro X, Roura-Poch P, Roca-Closa J, De Caralt-Mestres E (2017). Is the severe pain after Milligan-Morgan hemorrhoidectomy still currently remaining a major postoperative problem despite being one of the oldest surgical techniques described? A case series of 117 consecutive patients. Int J Surg Case Rep.

[7] Eroglu A, Apan A, Erturk E, Ben-Shlomo I (2015). Comparison of the anesthetic techniques. Sci World J.

[8] Formiga FB, Magi JC, Fernandes B, Boarini LR, Oliveira PD, Vieira RB (2017). Coloproctology the use of local anesthesia and sedation in transanal hemorrhoidal dearterialization with Doppler. J Coloproctol.

[9] Kushwaha R, Hutchings W, Davies C, Rao NG (2008). Randomized clinical trial comparing day-care open haemorrhoidectomy under local versus general anaesthesia. Br J Surg.

[10] Kotze PG, Junior IF, Freitas CD, Diniz F, Steckert-Filho Á (2011). Analysis of direct costs of anesthesia-related materials between spinal and venous anesthesia with propofol associated with local perianal block in hemorrhoidectomy. J Coloproctol.

[11] Ribotsky BM, Berkowitz KD, Montague JR (1996). Local anesthetics: is there an advantage to mixing solutions?. J Am Podiatr Med Assoc.

[12] Bhattacharyya S, Bisai S, Biswas H, Tiwary MK, Mallik S, Saha SM (2015). Regional anesthesia in transurethral resection of prostate (TURP) surgery: a comparative study between saddle block and subarachnoid block. Saudi J Anaesth.

[13] Kunitake H, Poylin V (2016). Complications following anorectal surgery. Clin Colon Rectal Surg.

[14] Hewitt-Smith A, Bulamba F, Ttendo S, Pappenheim K, Walker IA, Smith AF (2018). A mixed-methods evaluation of the Association of Anaesthetists of Great Britain and Ireland Uganda Fellowship Scheme. Anaesthesia.

[15] Zhong B (2009). How to calculate sample size in randomized controlled trial?. J Thorac Dis.

[16] Dakubo JCB, Bediako-Bowan AA, Nsaful J, Ampofo A (2015). Day case haemorrhoidectomy under local anaesthesia and conscious sedation. Int J Clin Med.

[17] Ellen S. Slovin’s formula sampling techniques. Sciencing. Consulted on 25th September 2021. 2018. Available at: https://sciencing.com/what-type-of-sample-is-used-for-probability-12749675.html.

[18] Kumar A, Aggarwal M, Singla R, Kansal T, Goyal S (2017). Open (Miligan Morgan) haemorrhoidectomy versus stapled haemorrhoidopexy: a comparative study. Br J Med Med Res.

[19] Senekal MG (2012). The safe spinal anaesthetic. Contin Med Educ.

[20] Jinjil K, Dwivedi D, Bhatnagar V, Ray RK, Tara S (2018). Perianal block: is it as good as spinal anesthesia for closed hemorrhoidectomies?. Anesth Essays Res.

[21] Mann CV (2002). Management of haemorrhoid complications. Thrombosis, fissure-in-ano, recurrence. Surgical Treatment of Haemorrhoids.

[22] Marshall SI, Chung F (1999). Discharge criteria and complications after ambulatory surgery. Anesth Analg.

